# In 2024, the *amyloid-cascade-hypothesis* still remains a working hypothesis, no less but certainly no more

**DOI:** 10.3389/fnagi.2024.1459224

**Published:** 2024-09-04

**Authors:** Christian Behl

**Affiliations:** The-Autophagy-Lab, Institute of Pathobiochemistry, University Medical Center of the Johannes Gutenberg University Mainz, Mainz, Germany

**Keywords:** Alzheimer, amyloid, APOE4, autophagy, GWAS, neurodegeneration

## Abstract

The *amyloid-cascade-hypothesis* of the pathogenesis of Alzheimer’s disease (AD) was introduced 32 years ago, in 1992. From early on, this clear and straight forward hypothesis received a lot of attention, but also a lot of substantial criticism. Foremost, there have always been massive doubts that a complex age-associated disorder of the most intricate organ of the human body, the brain, can be explained by a linear, one-dimensional cause-and-effect model. The amyloid-cascade defines the generation, aggregation, and deposition of the amyloid beta peptide as the central pathogenic mechanism in AD, as the ultimate trigger of the disease, and, consequently, as the key pharmacological target. Certainly, the original 1992 version of this hypothesis has been refined by various means, and the ‘formulating fathers’ followed up with a few reappraisals and partly very open reflections in 2002, 2006, 2009, and 2016. However, up until today, for the supporters of this hypothesis, the central and initial steps of the cascade are believed to be driven by amyloid beta—even if now displayed somewhat more elaborate. In light of the recently published clinical results achieved with anti-amyloid antibodies, the controversy in the field about (1) the clinical meaningfulness of this approach, (2) the significance of clearance of the amyloid beta peptide, and last but not least (3) the relevance of the amyloid-cascade-hypothesis is gaining momentum. This review addresses the interesting manifestation of the amyloid-cascade-hypothesis as well as its ups and downs over the decades.

## Introduction

According to the dictionary, a cascade is “something arranged or occurring in a series or in a succession of stages so that each stage derives from or acts upon the product of the preceding” (Merriam-Webster, 2024). Exactly such a series of pathogenic stages was proposed in the 1990’s for the pathogenesis of Alzheimer’s disease (AD). The amyloid-cascade-hypothesis was introduced in 1992 (Hardy and Higgins, 1992), and for decades of AD research to follow—even up until today—amyloid beta peptide, its generation, aggregation, and deposition has been the research focus for a larger part of the AD field. A linear and one-dimensionally directed sequence of events as represented by the amyloid-cascade, however, is strongly contrasted by our increasing knowledge about the mixed pathologies of neurodegenerative disorders, the many common pathways that different brain disorders share, and the multifactorial and multigenetic background of age-related neurodegeneration. Knowing all that certainly mandates a more holistic look at the events leading to dementia and AD, a perspective that considers and integrates a wider array of pathways that may include or exclude some role of amyloid beta deposition. The conceptual linear cause-and-effect view put forward by the amyloid-cascade is contrasted by many facts but is, for instance, explicitly exemplified by a more recent review by Morgan et al. (2022) concluding that “most pathways can be related to the pathogenesis of Alzheimer’s disease.” A key statement in this work is the conclusion: “We simply do not understand the disease well enough” (Morgan et al., 2022), which is still true in 2024. This work, together with hundreds of other original articles and reviews, emphasizes the multifactorial origin of AD (for a complete recent collection see Behl, 2023). Along that line of reasoning and after yet another round of anti-amyloid therapy failures, in 2022, the key author of the 1992 amyloid-cascade-hypothesis publication, John Hardy, stated, “…when we found amyloid mutations I thought, and the field thought, that sorting out amyloid was to sort out dementia. We had this idea of a magic bullet. We do not think that anymore” (Lourenco and Hausmann, 2022).

A plethora of data, models, and theories on the pathogenesis of AD was overruled by the consistent dominance of the amyloid-cascade-hypothesis since its introduction. Possible reasons for that are numerous and the development of AD research over the decades since the early introduction of the label “Alzheimer’s Disease” were presented and discussed extensively in a recent book (Behl, 2023). Central parts of this review are based on that publication and are, therefore, a personal opinion in larger parts, motivated by the recently developing fierce controversy about the published results of clinical studies with two amyloid-removing antibodies (donanemab and lecanemab). The fact is that almost all clinical approaches targeting amyloid beta peptide, its generation, its aggregation, and its removal have failed over the last three decades (Panza et al., 2019). The demonstrative satisfaction expressed by parts of the AD field referring to the published effects calculated from the clinical studies with the amyloid-clearing antibodies lecanemab and donanemab (van Dyck et al., 2023; Sims et al., 2023) may appear as narrowed and selected perception, and partly as wishful thinking. It actually appears like a continuation of the narrowed view on the pathogenesis of AD introduced by the concept of the existence of an amyloid-cascade that leads to AD.

## The *amyloid-cascade-hypothesis* emerges in the early 1990s

Up until today, many researcher in the AD field still promote the amyloid-cascade concluding that amyloid beta peptide is *the* key target in the search for an AD therapy. This view has already dominated the field over decades despite the fact that its promises and expectations were never fulfilled when translated into clinical application (Panza et al., 2019; Herrup, 2021; Behl, 2023; Granzotto and Sensi, 2024). For the supporters of the cascade, this has significantly changed in the last 2 years, i.e., since the publication of results of clinical studies on amyloid-removing antibodies and their effects, arguing that developed based on the cascade, we finally have a disease-modifying therapy in our hands that should be applied and further refined. However, as controversial as the amyloid-cascade-hypothesis was right from the beginning, as controversially discussed are the significance and relevance of the clinical studies’ results now, especially regarding their clinical meaningfulness, and the therapy’s potential (severe) side effect, costs, etc. (Walsh et al., 2022; Schneider, 2023; Kurkinen, 2023; Granzotto and Sensi, 2024). The massive limitations of application, efficacy, and possible problems (e.g., side effects) that come with the use of these antibodies are beyond the scope of this discussion and are covered elsewhere (Plascencia-Villa and Perry, 2023). Supporters of the current anti-amyloid therapy approach frequently point toward the already granted approval for lecanemab by the FDA (full approval in July 2023). However, even the approval procedure itself, and the general discussions around the drug initiated hot debates and controversies (see, e.g., Reardon, 2023).

A part of the AD community was curiously awaiting the approval of donanemab (actually it was approved by the FDA on July 2nd 2024) and is very optimistic that amyloid removal is at the core of a disease-modifying therapy (Boxer and Sperling, 2023; Selkoe, 2024; Jack et al., 2024); some go even further and consider the long-awaited positive clinical results as the final proof of the amyloid-cascade-hypothesis. Other parts of the AD field remain skeptical and critically regard the clinical results as a rather biased over-interpretation (Walsh et al., 2022; Kepp et al., 2023; Høilund-Carlsen et al., 2024a,b; Widera, 2024). Taken together, today, the AD field appears highly divided into at least two groups. While the supporters of the amyloid cascade concept regard the removal of amyloid as key to a future successful causal therapy, those who accept AD as a multigenetic and multifactorial age-related brain disease, consider amyloid deposition to be a late-step in the disease, or as a by-product and epiphenomenon.

**Figure 1:**
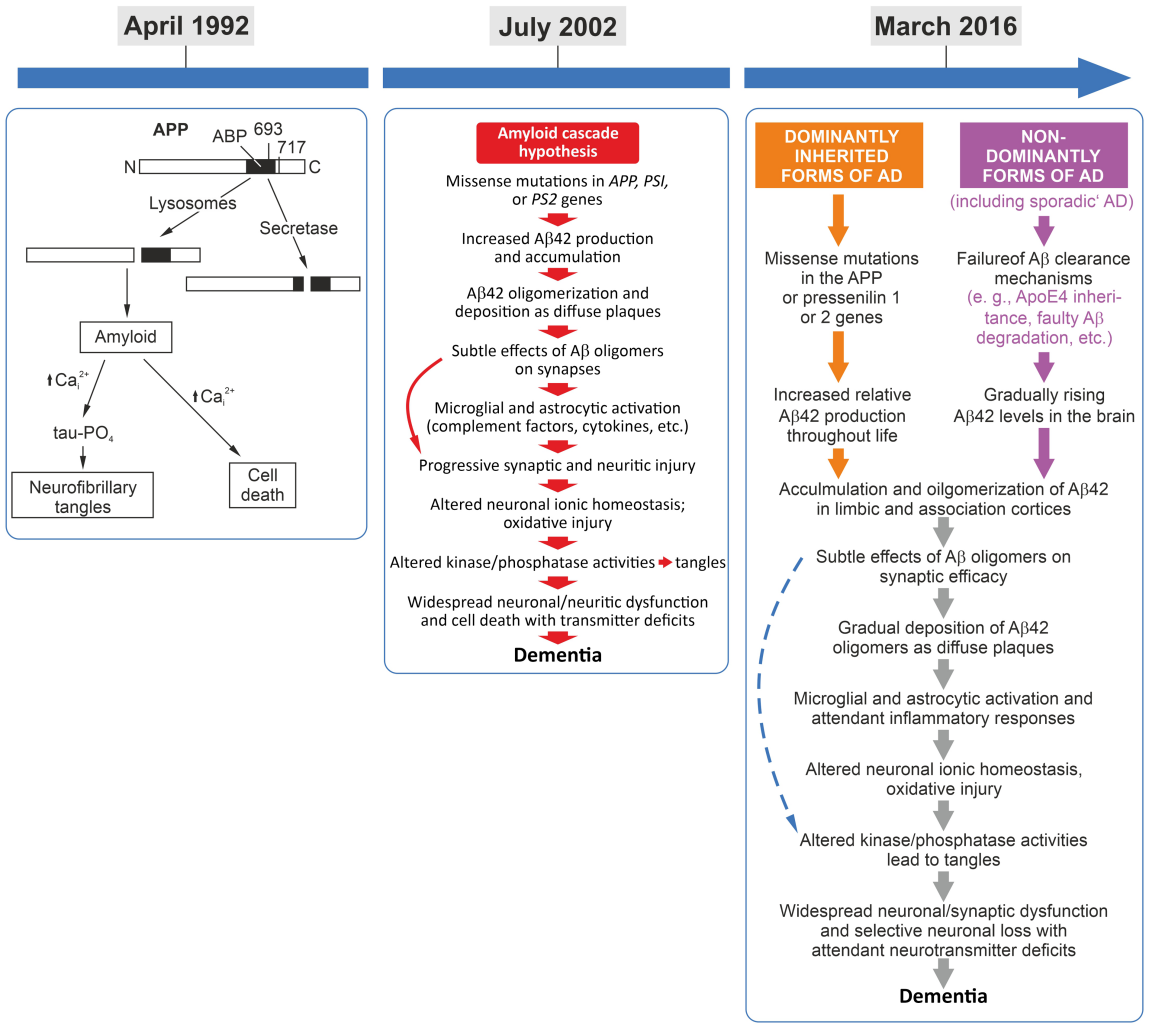
Left panel: April 1992, the amyloid-cascade-hypothesis was introduced and the term was coined (Hardy and Higgins, 1992). Middle panel: July 2002, the 10th anniversary of the cascade and the presentation of its refined version (Hardy and Selkoe, 2002). While some refinements were implemented, the basic linear flow regarding amyloid beta as first in the cascade still remained key to this pathogenesis model. Interestingly, tau-driven tangles were addressed in this overview rather as a pathogenic side thought. Right panel: March 2016, the 25th anniversary of the cascade, presenting an almost identical sequence of pathological events but with the differentiation of familial and sporadic AD, converging in the identical pathogenetic sequence (Selkoe and Hardy, 2016).

In the early 1990s and even before, excellent neurodegeneration research was already investigating various pathways as being crucial for AD pathogenesis. However, the impressive amount of high-quality experimental data on amyloid-beta peptide biology, biochemistry, and genetics linking this peptide to AD models and pathology, when put together, served as a good fundament for the formulation of the amyloid-cascade-hypothesis. Having worked on this peptide and its role in neuronal cell survival myself at that time, I remember that –among others– key triggers and kick-off papers for an emerging interest in amyloid-beta peptide back then were landmark publications by Yankner and colleagues demonstrating a principal neurotoxic effect of certain amyloid-beta peptide fragments in cell culture (Yankner et al., 1989, 1990). For many in the field, including myself, these exciting findings were a strong motivation to look further into the potential neurotoxicity of amyloid-beta (e.g., Behl et al., 1992, 1994; Price et al., 1992). Yet, in retrospect, the toxicity data were just one end of the many routes supporting the idea of an amyloid-driven cascade process. Consequently, in 1992, by including many more discoveries on amyloid beta biology, the original amyloid-cascade-hypothesis was formulated (Hardy and Higgins, 1992; Figure 1). It needs to be mentioned that while the term “amyloid-cascade-hypothesis” was actually coined by the frequently cited 1992 paper of Hardy and Higgins, the basic concept of a cascade process was established in a total of three review articles: (1) “The molecular pathology of Alzheimer’s disease” by Selkoe published in Neuron in 1991 (Selkoe, 1991), (2) “Amyloid deposition as the central event in the etiology of Alzheimer’s disease” by Hardy and Allsop, published in Trends in Pharmacological Sciences in 1991 (Hardy and Allsop, 1991), and (3) “Alzheimer’s disease: the amyloid cascade hypothesis” by Hardy and Higgins, published in Science in 1992 (Hardy and Higgins, 1992). Many researchers in the AD field regard this collection of papers as the “start of the dominance of the amyloid-cascade-hypothesis” (Hardy, 2006).

## The *amyloid-cascade-hypothesis* is challenged from early on

Concerns about the amyloid-cascade concept were already raised shortly after its first presentation. Inconsistencies and weak points in its interpretation as well as its overall validity was questioned prominently in 1998 (Neve and Robakis, 1998). It is necessary and a key scientific procedure to challenge a scientific working hypothesis; formally, it is the core of epistemology, practically, it is the guarantee for scientific progress. A hypothesis that can resolve all criticism on the basis of experimental data achieved by appropriate methods will, in the end, come out stronger. If concerns cannot be refuted, the presented hypothesis needs to be significantly changed or withdrawn; there are many examples in the history of science and epistemology of this process (summarized in, e.g., Jacobs and Theunissen, 2022; Behl, 2023). While this overview outlines some key stations of the evolution of the amyloid-cascade-hypothesis over the past three to four decades, including its dominance and refinements, a number of more extensive critical analyses are available elsewhere (e.g., Smith and Perry, 1998; Robinson and Bishop, 2002; Lee et al., 2004a; Whitehouse and George, 2008; Kern and Behl, 2009; Herrup, 2015; Morris et al., 2014; Lock, 2013; Behl, 2017; Robakis, 2020; Herrup, 2021; Behl, 2023).

From early on, AD has mostly been handled as a plaques-and-tangles disease, fueled by Alois Alzheimer’s initial microscopic and histopathological observation that, besides other (!) alterations, disease tissue exhibits plaques and tangles. One may speculate that the subsequent interpretation of Alzheimer’s report from 1907 has, since then, been highly selective. Similar subsequent histopathological findings further hardened the case for plaques (and tangles) as the cause of the disease. It is clear today that pure AD characterized by the occurrence of plaques and tangles (i.e., amyloid beta and tau protein) is very rare and most AD (and dementia) cases show mixed histopathologies, including different types of aggregated proteins seen also in other neurodegeneration scenarios. In fact, the presence of mixed pathologies in many cases characterizes AD as it is considered based on today’s knowledge (Rabinovici et al., 2017; Jellinger, 2022).

Although the amyloid-cascade-hypothesis overshadowed the AD research in the 1990s and controlled the narrative in the field, there was never a shortage of alternative ideas regarding the causes of neurodegeneration in AD, aside from amyloid-beta. However, the majority of the alternative concepts, some of which I will shortly introduce later, mostly lacked a simple linear cause-and-effect relationship. They took a wider perspective on the biology of nerve cells, brain aging, and the various pathways involved in neuronal function, survival, and degeneration. About 10 years after its introduction, a series of articles summarized key points of concern regarding the validity of the amyloid-cascade. For instance, in 2002 some in the field raised substantial doubts on the pathological conclusions made by the amyloid-cascade concept. As mentioned, numerous reviews effectively outlined these concerns; one of them focused on highlighting “inconsistencies between the predictions of the amyloid hypothesis and the published data” (Robinson and Bishop, 2002). Two years later, in 2004, Mark Smith and colleagues summarized their critics shared by many about the one-sided view on AD pathogenesis as presented in the amyloid-cascade: “In fact, the Amyloid Cascade Hypothesis has come to dominate the field both in terms of proposed disease mechanism as well as potential for therapeutic intervention” (Lee et al., 2004b). The authors addressed substantial inconsistencies in the prominent hypothesis, and looked more closely into pathology, cell biology/biochemistry, and genetics.

In their publication, Lee and colleagues argue that the presence of tau tangles (neurofibrillary tangles) together with the loss of neurons is more strongly correlated with cognitive decline than amyloid beta peptide depositions (amyloid plaques). Interestingly, back then research already revealed that amyloid deposits are also frequently found in non-demented elderly individuals, rendering amyloid deposition in the brain a rather concomitant epiphenomenom. We know today that 30–40% of cognitively normal elderly individuals exhibit amyloid deposition in their brains, which would fulfill the histological requirements of an AD pathology. This fact has currently initiated another hot debate on the proposed biological definition of AD (based on protein changes in amyloid and tau) called the recently revised AT(N)-system with A for amyloid, T for tau (and N for neurodegeneration) as justification (Høilund-Carlsen et al., 2024b; Lista et al., 2024; Jack et al., 2024).

Lee and colleagues further scrutinized the cell biological and biochemical aspects summarized by the amyloid-cascade. They argue that, rather than being a product of a pathophysiological process, amyloid beta peptide is, in their view, a regular product of the cell’s physiological metabolism. Moreover, they point out that, despite the manifold presented convincing *in vitro* neurotoxic effects of amyloid beta protein, the *in vivo* toxicology remains controversial. “[W]hile cell culture ‘models’ were key in formulating the Amyloid Cascade Hypothesis, they do not appear to be an accurate reflection of any *in vivo* or diseased conditions” (Lee et al., 2004b). For these colleagues, as for many others in the field, the generation and release of amyloid beta represents an adaptive and protective response to various brain insults. Interestingly, with their first definition of the amyloid-cascade-hypothesis Hardy and Higgins already suggested that “other causes of Alzheimer’s act by initially triggering [amyloid beta peptide] deposition,” and that “there is an association between head trauma and Alzheimer’s” (Hardy and Higgins, 1992). As further support of the amyloid-beta-production-as-a-stress-response idea, the same authors suggested that “amyloid deposition occurs as an acute response to neuronal injury in both, man and animals” and that “APP increases in response to a number of neuronal stresses” (Hardy and Higgins, 1992). One may wonder why these valid concerns and explicitly presented ideas and alternative interpretations of AD’s pathogenesis have remained largely unheard and did not lead to a significant repositioning of AD research in the time after 1992.

The favored role of amyloid beta peptide was further cemented by genetic findings that, to date, are considered as strong pillars in support of the function of amyloid beta in AD development. After the introduction of the amyloid cascade, interestingly, autosomal dominant mutations in the genes encoding the amyloid precursor protein (APP), presenilin 1 (PSEN1), and presenilin 2 (PSEN2) were identified. These mutations are associated with early onset familial AD, and a variety of *in vitro* (and later also *in vivo* mouse transgenic) studies could show that they trigger the generation and release of increased levels of amyloid beta peptide. I would like to emphasized here that these genetic and biochemical data on inherited/familial forms of Alzheimer’s Disease are really convincing, the question is, are they relevant for all types of AD, meaning also for the non-genetic strictly age-related forms? It is necessary to stress that these familial early-onset forms of the disease (based on *APP*, *PSEN1*, or *PSEN2* mutations) are extremely rare; they are in the range of a prevalence of under 5% of all AD cases. Although the data on familial AD genes are strong, are well acknowledged and make a good case, Lee et al. (2004b) introduced an alternative interpretation by pointing out that “while one interpretation of the available data is that ‘mutation leads to increased Aβ leads to disease’ an equally valid explanation is that ‘mutation leads to disease leads to increased Aβ’.” This alternative perspective suggested that amyloid beta might stand at the end of the mutation-driven process rather than at the beginning. Lastly, we need to acknowledge that the majority of AD cases are strictly age-related and sporadic in nature (senile dementia) pointing toward a multifactorial origin in the majority of AD cases. In retrospect, the discovery of the APP, PSEN1, and PSEN2 mutations causing early-onset familiar AD was of key importance for the support of the amyloid-cascade and its future dominance. Notably, these mutations linked to early onset AD reflect the historic fact that almost all histopathological cases found in the initial phase of research after the coining of the term “Alzheimer’s Disease” were derived from young individuals (early onset, presenile dementia). Nevertheless, decades after Alzheimer and colleagues first described such cases of early onset dementia and actually well before the discovery of AD-associated mutations, already in 1976, the field of AD research began a complete narrowing of its focus.

Largely based on histopathological similarities, a short editorial by Katzman (1976) proposed to combine the etiopathology of the very rare familial genetic forms associated with presenile dementia with the incomparably vast number of age-associated sporadic (idiopathic, senile dementia) forms of the disease. Katzman’s view that AD is a “major killer” was based “on the assumption that Alzheimer disease and senile dementia are a single process and should, therefore, be considered a single disease” (Katzman, 1976). This move was fundamental in directing the future of AD research, blinding out the multifactorial origin of the majority of AD cases (senile dementia) and further cementing the narrow focus on amyloid beta biology.

Today, genetics tells us that there are dozens of (genetic) risk factors that may influence the onset and course of AD. So-called human genome-wide-association studies (GWAS) have presented us a novel landscape of AD genetics. The identified genetic risk factors can be assigned to a variety of potentially relevant pathomechanisms besides APP metabolism, including innate immunity, lipid metabolism, endosome-lysosome activity, ubiquitin proteasome and synaptic function, cholesterol metabolism, ephrin signaling, and complement activation (Bellenguez et al., 2022; see also Niagads Data Sharing Service). In conclusion, today, genetics significantly support the multigenetic and multifactorial nature of AD.

Around the same time the concept of the amyloid-cascade emerged, the field had also developed valid alternative approaches, most of which were largely neglected at the time; I would like to shortly highlight some of them here.

Efficient intracellular homeostasis is the key to cellular function and survival, in particular in non-mitotic cells such as neurons. Over their entire lifetime, in particular non-dividing cells, neurons, have to deal with a variety of ongoing damage and repair problems on a single-cell basis. Certainly, the need for providing permanent DNA repair is one key challenge. Continuous disturbances of protein homeostasis (proteostasis; balance of generation, transport, folding, clearance of proteins) can lead to neuronal dysfunction and, eventually, to degeneration. Interestingly, a dysregulation of proteostasis and especially protein clearance as a possible cause of neurodegeneration was suggested from early on (Smith and Perry, 1994). Since a dysfunctional clearance of defective and aggregated proteins leading to the build-up of intracellular protein aggregates is detrimental to neurons, the detailed analysis of intraneuronal protein degradation pathways were an important focus in the 1990s (Nixon and Cataldo, 1994; Nixon et al., 2000). Subsequently, the concept of dysfunction of the endosomal-lysosomal pathway in neurons was introduced and certainly represents a valid alternative to explain the pathogenesis of AD. Although the experimental data supporting the view that an endosomal-lysosomal defect is the prime basis for AD-associated neurodegeneration were published in the 1990s, it took more than 10 years to receive the appropriate attention in the neuroscience community. In 2010, this alternative concept was presented to a wider audience of neuroscientists at the Society for Neuroscience in 2010 and subsequently summarized (Pimplikar et al., 2010): “…a growing body of evidence shows that Aβ peptides are unlikely to be the sole factor in AD etiology. Evidence that Aβ/amyloid-independent factors, including the actions of AD-related genes, also contribute significantly to AD pathogenesis was presented in a symposium at the 2010 Annual Meeting of the Society for Neuroscience. Here we summarize the studies showing how amyloid-independent mechanisms cause defective endo-lysosomal trafficking, altered intracellular signaling cascades, or impaired neurotransmitter release and contribute to synaptic dysfunction and/or neurodegeneration, leading to dementia in AD” (Pimplikar et al., 2010).

Another prominent example of a neglect of important AD-related facts in the 1990s is the lack of appreciation for a detailed pathogenetic role of the apolipoprotein E (APOE) in AD, based on the key finding that the presence of the isoform APOE4 is a fundamental risk factor for AD (Saunders et al., 1993). Despite its significance as an AD risk factor, the role of APOE4, upon discovery, was mainly investigated in the context of its relationship with amyloid beta peptide biology (if at all), further demonstrating the amyloid-centric direction of AD research in the 1990s. Fortunately, in recent years this has changed dramatically and there is consensus about a prominent pathogenic role for APOE and lipid transport, and even an “APOE Cascade Hypothesis” in AD was introduced (Martens et al., 2022; Bailey et al., 2024).

Taken together, despite its acceptance and clarity, the amyloid-cascade-hypothesis was controversially discussed right from the beginning. For a detailed and current pro and contra discussion of this topic, readers should consult available expert reviews (Herrup, 2015; Tse and Herrup, 2017; Herrup, 2021; Bermejo-Pareja and Del Ser, 2024).

## The *amyloid-cascade-hypothesis* experience refinements and reappraisals over the years

A decade after its introduction, the amyloid-cascade-hypothesis was refined and reassesed by its key advocates (Hardy and Selkoe, 2002). The first reappraisal published in *Science* summarized: “It has been more than 10 years since it was first proposed that the neurodegeneration in Alzheimer’s disease (AD) may be caused by deposition of amyloid β-peptide (Aβ) in plaques in brain tissue. According to the amyloid hypothesis, accumulation of Aβ in the brain is the primary influence driving AD pathogenesis. The rest of the disease process, including formation of neurofibrillary tangles containing tau protein, is proposed to result from an imbalance between Aβ production and Aβ clearance” (Hardy and Selkoe, 2002). Interestingly, on the one hand, the publication openly defines a principal problem of the amyloid-cascade-hypothesis stating that “in considerable part, the amyloid hypothesis remains controversial because a specific neurotoxic species of Aβ and the nature of its effects on neuronal function have not been defined *in vivo*” (Hardy and Selkoe, 2002). On the other hand, other parts of this work further emphasizes the strength and the dominance of this hypothesis and of amyloid-centered research with statements such as: “none of the currently perceived weaknesses of the amyloid hypothesis provides a compelling reason to abandon this idea, although together they certainly point to important gaps on our understanding of AD” (Hardy and Selkoe, 2002).

Interestingly, in the 1990s, during the early stages of intense molecular research on the causes of AD, the dispute over whether amyloid (plaques) or tau (tangles) is responsible for disease development divided the AD community into two camps, “Baptists” and “Tauists,” until they were ‘pathomechanistically’ united in the flow of the amyloid cascade of events (Lewis et al., 2001; Götz et al., 2001; Lee, 2001; Mudher and Lovestone, 2002). While the amyloid-cascade had now adopted a role for tau as a downstream component of the sequence of events, it also reinforced that amyloid beta initiates the process. In addition, the authors discussed potential paths and strategies toward a treatment, recommending AD research directions that strongly concentrate on amyloid beta biology. They suggested (1) searching for inhibitors of beta- and gamma-secretases, in order to block amyloid beta generation and (2) finding inhibitors of amyloid beta aggregation and (3) developing measures to clear it from the brain. In addition, this ‘amyloid-cascade-revisited’ considered anti-inflammatory approaches and the modulation of cholesterol homeostasis, as well as scavenging of metal ions, and, in a more general manner, targeting synapses and neurodegeneration as a whole.

Taken together, this 10-years-after reappraisal significantly developed the amyloid-cascade-hypothesis by considering a broader bandwidth of additional, potentially relevant pathogenic factors. Yet, the role of amyloid beta peptide as the initial trigger, the beginning, of the pathologic cascade in AD was never questioned. Pointing—in their view—to a lack of alternatives, the authors finally concluded that “[s]everal perspectives on the deficiencies of the hypothesis have been put forward […], but an alternative hypothesis explaining the cause and early pathogenesis of AD that has as much experimental support as the Aβ hypothesis has not emerged” (Hardy and Selkoe, 2002). After presenting only two of the many neglected yet potentially important additional perspectives on AD’s pathogenesis, one may disagree with this strong statement. Another—as I find-—very interesting aspect of this 2002 review is the fact that the cascade displayed in its summary figure begins with “missense mutations in APP, PS1, or PS2 genes.” However, in the legend, this is described as “the sequence of pathogenic events leading to AD proposed by the amyloid cascade hypothesis” with no further differentiation of the familial (genetic, early-onset) and non-familial (age-associated, late-onset) forms. In my view, this presentation insinuates that the presented cascade of events is one-to-one transferable to the age-associated sporadic forms of AD, reinforcing also Katzman’s statements form 1976. Nevertheless, further reappraisals, refinements, and appreciations of the amyloid-cascade concept kept following in shorter time intervals.

In the same year, i.e., 2002, John Hardy published another comment entitled “Testing times for the ‘amyloid cascade hypothesis” (Hardy, 2002). Interestingly, this was a more open interpretation including some cautionary perspectives on the hypothesis: “It is not a prediction of the amyloid cascade hypothesis that the amount of amyloid deposition would correlate with the degree of dementia. I have always regarded such correlative studies as almost a complete waste of time because they rely on the credulous supposition that pathology waits to be counted” (Hardy, 2002). Further, he states *expressis verbis* that “[t]he question of whether the amyloid cascade hypothesis is correct is a complex one. It is complex because each [of] its proponents has a marginally different and (one hopes) evolving view of the hypothesis and also because, given the huge number of uncertainties, it is clearly only ever going to be an approximation to the truth. Certainly my view of the disease pathogenesis has changed over the last 10 years but in an evolutionary rather than a revolutionary way. Do we need a revolutionary change in our thinking about the disease? I would say not yet” (Hardy, 2002). Certainly, when considering the development of the amyloid-cascade-hypothesis over the last 30 years, by 2002—i.e., 10 years after its first introduction—it had already been expanded by additional pathogenetic factors to be considered (Figure 1). However, the linearity of cause (amyloid) and effect (dementia) was still maintained and confirmed as it was in 1992.

Reading the 2006 recap of the 1992 amyloid-cascade is truly remarkable (Hardy, 2006). This work increased the number of fathers and mothers of the original hypothesis and acknowledged the significant impact of a number of other scientists’ work. John Hardy also gave insights into the making-of of the 1992 *Science* paper, which—when read today—is truly astonishing, knowing that this very paper was the igniting spark of an entire research field (Alzheimer’s amyloid biology) that has dominated the field to date. Apparently, “the review took possibly a week to write” (Hardy, 2006). And one may feel puzzled reading that “the article in *Science* was intended to generate ideas and act as a framework for a research agenda, not to be a definitive statement,” and “I certainly did not mean it to be laid down on a tablet of stone and consulted to ascertain ultimate wisdom about Alzheimer’s disease” (Hardy, 2006). As mentioned, this 2006 reconsideration about the actual formulation of the influential amyloid-cascade-hypothesis appeared open, reflected, and also self-critical exemplified also by the remark: “I have found it irritating to be asked time and time again to present and defend it” (Hardy, 2006).

Yet, another 3 years later, in 2009, the next reconsideration of the hypothesis was presented, “The amyloid hypothesis for Alzheimer’s disease: a critical reappraisal” (Hardy, 2009). One may speculate about the motivation to publish it at this very time, but it could partly be due to the increasing pressure on the Alzheimer’s amyloid field because of the ongoing lack of an effective disease modifying (causal) therapy based on the amyloid-cascade-hypothesis. “Increasingly over the last 3 years, there has been a chorus of concern that the amyloid hypothesis was not delivering effective therapies for the disease. Whether this chorus is like the dawn chorus, heralding a bright new era of Alzheimer research, or a malcontent’s chorus, merely whingeing that their grants go unfunded, is open to debate. As one of the two (inaccurately) credited with originating the amyloid hypothesis (Selkoe, 1991; Hardy and Allsop, 1991; Hardy and Higgins, 1992; Hardy and Selkoe, 2002), in this review, I offer my opinion” (Hardy, 2009 and references therein).

This work of 2009 presents an open discussion and validation of previously made predictions of the amyloid cascade. One should remember that by then, three anti-amyloid clinical trial concepts had all failed (active immunization, passive immunization, modulators of gamma-secretases). The publication also referred to two views as alternatives to the amyloid-cascade-hypothesis, namely the “presenilin inhibition hypothesis” and the “second hit hypothesis.” Moreover, it also pointed out that there was a lack of sufficient knowledge about the role of the protein the amyloid beta peptide is processed from, the “amyloid precursor protein” (APP). “It is surprising that, despite the fact that it is more than 20 years since the APP gene was cloned (Kang et al., 1987), we have very little idea of its function and almost no idea as to whether Aβ has a function or not. One reason for this lack of knowledge is that APP knockout mice have very little overt phenotype (Heber et al., 2000) but the major reason for this lack of knowledge is that this has not been a major research priority. We also have no idea as to whether Aβ has a function or not” (Hardy, 2009 and reference therein). Finally, this 2009 work suggested that “it makes sense to pursue other targets beyond amyloid beta (Noble et al., 2005)” (Hardy, 2009 and reference therein). I should mention that, of course, a huge number of publications authored by active members of the AD field addressed a possible role of amyloid beta peptide in AD, and presented many more as well as less critical reconsiderations of the amyloid-cascade (see also in Behl, 2023). The focus of the collection I present here is to chronologically display the development of the amyloid-cascade-hypothesis, its refinements, but also its critical reflections by its key protagonists and promoters.

On the occasion of the 25th anniversary a review article—“The amyloid hypothesis of Alzheimer’s disease at 25 years”— was published (Selkoe and Hardy, 2016). Looking at the framework and how it is displayed, one can concluded that this work extended the cascade only marginally as compared to the cascade presented 12 years ago on the occasion of its 10th anniversary (Figure 1). It is eye-catching that in the figure summarizing “[t]he sequence of major pathogenic events leading to AD” (Figure 1 in Selkoe and Hardy, 2016) the dominantly inherited/genetic forms of AD and the non-dominant/non-genetic forms of AD are displayed now as separate entries in the cascade. However, two steps further downstream, both separate entries (forms of AD cases) converge at the “[a]ccumulation and oligomerization of Aβ42 in limbic and association cortices” into the same subsequent cascade of pathological events” (Figure 1 in Selkoe and Hardy, 2016). This anniversary edition is a deep and excellent dive into the molecular findings made by AD-amyloid research since its kick-off in 1992. It points out the possible impact of additional pathogenetic factors, emphasizes “the need for alternative agents that target other early features of this complex and devastating syndrome,” and also, critically, the “pending issues,” such as: “What are the toxic species of Aβ and tau?” and “What is the connection between Aβ and tangle pathology? Is it direct and cell autonomous or does it involve non-neuronal cells?,” etc. (Selkoe and Hardy, 2016). Nevertheless, this work can also be interpreted as a supporting reappraisal and yet another strong reinforcement of the amyloid-cascade-hypothesis, as exemplified by the closing remark of its abstract: “Although many factors contribute to AD pathogenesis, Aβ dyshomeostasis has emerged as the most extensively validated and compelling therapeutic target” (Selkoe and Hardy, 2016). One year after this landmark anniversary review, however, a paper by Hardy (2017) on “the discovery of Alzheimer-causing mutations in the APP gene and the formulation of the ‘amyloid-cascade-hypothesis’” displayed a partially new spin, and, again, was rather reflective. The focus of this reconsideration was, one the one hand, to remind readers of the competitive efforts of the different AD labs that did the initial genetic analyses aimed at linking APP mutations to AD. On the other hand, it also referred to the zeitgeist, openly pointing out “[e]rrors and excitement in AD” that occurred in that “feverish” research atmosphere around 1987 (Hardy, 2017). Here, Hardy states: “I had always thought of genetics as an independent way of testing hypotheses of causation. There had been many competing theories of AD and I simply believed that genetics would allow a decision about these competing theories to be made. Genetic analysis told us that amyloid was the cause of AD in these families, and also in Down syndrome. Without much thought, I wrote out my verdict on this work first with David Allsop and then with Gerry Higgins (Hardy and Allsop, 1991; Hardy and Higgins, 1992). Contemporaneously, Dennis Selkoe came to the same conclusion (Selkoe, 1991), and these three papers, which Dennis and I have subsequently updated (Hardy and Selkoe, 2002; Selkoe and Hardy, 2016), form the basis for the amyloid hypothesis of the disease. Together these papers have been cited more than 10,000 times” (Hardy, 2017 and references therein). Again, reading this review may leave one somewhat puzzled and pensive, especially considering a final self-critical and honest thought: “A third lesson: given all the mistakes that we and others made in the hotheaded analyses of 1987, is try not to be swept along. Speaking for myself, and also I suspect for the other groups involved in the *Nature* and *Science* papers in that year: we were too fast to be careful and, I suspect, the journal editors and reviewers were equally careless” (Hardy, 2017).

Nevertheless, the openness and the self-reflection, as presented during the evolution of the amyloid-cascade-hypothesis, has to be appreciated. In fact, literature gives us many more examples in which proponents of the amyloid cascade acknowledge and openly admit that there might be alternative factors involved as cause of AD. However, so far, such doubts and reservations have apparently not become strong enough to trigger a *paradigm shift* in AD research in larger parts of the AD field, and the main and narrow research focus on amyloid beta currently continues. Naturally, one of the strongest arguments driving the critics on concentrating exclusively on amyloid beta peptide came from the recurring fact that for about 30 years anti-amyloid approaches failed clinically (Panza et al., 2019; Castellani et al., 2019). Expectations and pressure on the clinical trials were immense and so was the frustration upon the failures afterwards. This bi-polar situation of high hopes and deep fall can be observed over the last three to four decades of AD research (for detailed discussion, see Behl, 2023). More recently, some parts of the AD field have uplifted the reported results of clinical anti-amyloid approaches (van Dyck et al., 2023; Sims et al., 2023) to the final proof of the amyloid-cascade-hypothesis, igniting the next stage of controversy on this dominating hypothesis. Seeing also the most recent controversial debate of the published clinical results on the use of the anti-amyloid antibodies lecanemab and donanemab, this dispute might be ongoing and not be resolved in the near future. The amyloid-cascade-hypothesis appears revitalized by the antibody performance in affected individuals (with a focus on early AD cases). Currently, the field experiences a critical phase again, and for some, the glass is half-full, for others, it is half-empty—or not even that. An opinion frequently shared in a constantly increasing number of publications is that “we simply do not understand the disease well enough” (Morgan et al., 2022) and I would agree on that, seeing that our understanding of the complexity of the brain itself is so limited (Shapson-Coe et al., 2024). A call for a *paradigm shift*, which I think is long overdue, has long been demanded by many in the field, and has actually been proposed by several recent discussions (e.g., Jacobs and Theunissen, 2022; Herrup, 2021; Behl, 2023). The required shift of a scientific paradigm will not inevitably lead to ignoring amyloid beta peptide as one aspect of AD but, rather, will alleviate the pressure on it being the only possible initial trigger of the disease; amyloid beta peptide may be entering the picture later in the disease process but not at the beginning. A more detailed discussion on the alternative roles of amyloid beta in AD was summarized elsewhere (Behl, 2023).

Seeing all the available data on amyloid-beta of the last 30 years, it is of course understandable to stick to the amyloid-cascade as the basis of a *working hypothesis*, to conclude significant support (or even proof) from recent clinical data, and continue further developing the hypothesis as a disease concept for another 10 years or longer. No one can predict what the title of a future review, perhaps on the occassion of the 40th or 50th anniversary of this hypothesis, will be. However, in my view, to better understand and define AD, and to get closer to effective prevention and therapy, the research focus needs to be significantly expanded as soon as possible.

Interestingly, the amyloid-cascade-hypothesis is a prime example for an idea presented by the philosopher Thomas S. Kuhn on trajectories of scientific paradigms (Kuhn, 1970). Kuhn’s discerns three key phases in science and in the evolution of scientific progress: (1) the preparadigmatic phase of science (competing views and approaches exist), (2) the paradigmatic phase of science (one particular paradigm dominates field), and (3) the revolutionary phase (certain inconsistencies and anomalies trouble the current theory, which is attacked and under pressure; novel views appear). This ultimately culminates in a paradigm shift and a scientific revolution. Regarding the amyloid-cascade-hypothesis, I would say, we are not quite there yet; we have not reached the final steps of stage 3, the necessary *paradigm shift*. Very likely, it will take more time to get there, and the amyloid-cascade will continue to be the basis of many basic, preclinical, and clinical research which is already ongoing or planned. Yet, in my personal view and as written in the headline of this discussion, in 2024, the amyloid-cascade-hypothesis still remains a working hypothesis—no less but certainly no more.

## Data Availability

The original contributions presented in the study are included in the article/supplementary material, further inquiries can be directed to the corresponding author.
